# Pulsed Vacuum Arc Deposition of Nitrogen-Doped Diamond-like Coatings for Long-Term Hydrophilicity of Electrospun Poly(ε-caprolactone) Scaffolds

**DOI:** 10.3390/membranes12111080

**Published:** 2022-10-31

**Authors:** Semen Goreninskii, Yuri Yuriev, Artem Runts, Elisaveta Prosetskaya, Elizaveta Sviridova, Evgenii Plotnikov, Ksenia Stankevich, Evgeniy Bolbasov

**Affiliations:** 1 B.P. Veinberg Research and Educational Centre, Tomsk Polytechnic University, 634050 Tomsk, Russia; 2 Onconanotheranostics Laboratory, Shemyakin-Ovchinnikov Institute of Bioorganic Chemistry RAS, 117997 Moscow, Russia; 3 Microwave Photonics Lab, V.E. Zuev Institute of Atmospheric Optics SB RAS, 634055 Tomsk, Russia; 4 Research School of Chemistry & Applied Biomedical Sciences, Tomsk Polytechnic University, 634050 Tomsk, Russia; 5 Chemistry and Biochemistry Department, Montana State University, Bozeman, MT 59717, USA

**Keywords:** diamond-like coatings, electrospun scaffolds, polycaprolactone, biomaterials

## Abstract

The surface hydrophobicity of poly(ε-caprolactone) electrospun scaffolds prevents their interactions with cells and tissue integration. Although plasma treatment of scaffolds enhances their hydrophilicity, this effect is temporary, and the hydrophobicity of the scaffolds is restored in about 30 days. In this communication, we report a method for hydrophilization of poly(ε-caprolactone) electrospun scaffolds for more than 6 months. To that end, diamond-like coating was deposited on the surface of the scaffolds in a nitrogen atmosphere using pulsed vacuum arc deposition with sputtering of graphite target. This approach allows for a single-side hydrophilization of the scaffold (water contact angle of 22 ± 3° vs. 126 ± 2° for pristine PCL scaffold) and preserves its structure. With increased nitrogen pressure in the chamber, sp^3^-hybridized carbon content decreased twice (sp^2^/sp^3^ ratio decreased from 1.06 to 0.52), which demonstrates the possibility of tailoring the content of carbon in sp^2^ and sp^3^ hybridization state. Nitrogen content in the deposited coatings was found at 16.1 ± 0.9 at.%. In vitro tests with fibroblast cell culture did not reveal any cytotoxic compounds in sample extracts.

## 1. Introduction

Owing to their high biocompatibility, biodegradability and mechanical properties, electrospun poly(ε-caprolactone) (PCL) scaffolds are widely applied in biomedical engineering, tissue engineering and drug-delivery systems [1]. Surface hydrophobicity remains a significant drawback of these scaffolds, as it prevents their interactions with cells and tissues. To overcome this limitation, several physical and chemical PCL surface modification approaches have been developed [2]. Whereas chemical approaches are mainly based on covalent grafting of reactive groups by treating the scaffolds in various solutions [3], physical approaches utilize plasma treatment [4]. The advantage of chemical approaches is their relative simplicity; however, they do not allow for a single-side modification of the scaffold, which is important in the case of artificial grafts and vascular patches. In contrast, with plasma treatment side-specific modification is possible, but the effect is temporary (up to one month) [5]. This limits both the shelf life and application scope of the plasma-treated scaffolds, especially in clinical practice.

Diamond-like coatings (DLC) formed on the surface of metal and ceramic implants provide hydrophilic, antibacterial, osteogenic and anti-thrombogenic properties, enhancing their endothelization and hemocompatibility [6]. Therefore, we envisaged that the deposition of DLC coatings on the surface of electrospun PCL scaffolds would be an efficient approach to their functionalization. The following methods are currently used for the deposition of DLC coatings: ionized evaporation, magnetron sputtering, high-power impulse magnetron sputtering, filtered cathode vacuum arc, ion-beam deposition, arc-ion plating and laser-arc deposition [7]. However, the application of these methods for the modification of electrospun PCL scaffolds is limited due to structural failure of PCL scaffolds, with a melting temperature of 60 °C [8]. Pulsed vacuum arc deposition (PVAD) is a promising method for the deposition of DLC coatings on the surface of electrospun PCL scaffolds. This method allows for the generation of a carbon plasma with 40–90 eV ion energy, does not require an acceleration potential on the substrate and provides a high rate of condensate formation (up to 1 × 10^4^ Å/s). Importantly, the substrate temperature does not exceed 70 °C during deposition [9]. Moreover, with variation of the working gas pressure and composition, it is possible to tailor the properties of the deposited coating [10].

The deposition of DLC coatings on the surface of electrospun PCL scaffolds using PVAD has not been reported to date. The effects of coating deposition on scaffold structure, wetting, chemical composition, and biocompatibility remain unknown. Herein, we report the possibility of DLC coating formation on the surface of electrospun PCL scaffolds by the PVAD technique, as well as the key properties of the modified PCL scaffolds.

## 2. Materials and Methods

PCL scaffolds were manufactured using an electrospinning technique from a 9% PCL (80,000 g/mol, Sigma, Burlington, MA, USA) solution in trichloromethane (CHCl_3_) (Ekros, Moscow, Russia). The NANON-01A electrospinning setup (MECC CO., Fukuoka, Japan) was equipped with a 200 mm steel collector (⌀ = 100 mm), and polymer solution was supplied with a flow rate of 6 mL/h through an 18 G needle. The needle-to-collector distance was 190 mm, and the applied voltage was set at 20 kV, with a collector rotation speed of 50 rpm. For the removal of residual solvents, the fabricated scaffold was stored in a VD 115 vacuum furnace (Binder, Tuttlingen, Germany) at a temperature of 40 °C under a pressure of 0.1 Pa.

The DLC coatings were deposited using the pulsed vacuum arc deposition technique. High-purity (99.99%) graphite target and nitrogen (99.99%) were used. The deposition was performed under the following conditions: discharge voltage, 170 V; impulse duration, ~500 µs; current peak amplitude, 1500 A; 1 Hz impulse frequency; 3000 pulses per sample; target-to-substrate distance, 200 mm. Three groups of coatings were formed depending on the nitrogen pressure in the chamber: 5 × 10^−3^, 5 × 10^−2^ and 5 × 10^−1^ Pa. Non-coated scaffolds were used as a control group.

The scaffold morphology was investigated using scanning electron microscopy (SEM) (Tescan VEGA 3, Warrendale, PA, USA). The fiber diameter of the membranes was determined from the SEM images of 10 different fields of view using Image J 1.38 software (National Institutes of Health, Bethesda, MD, USA). To calculate an average diameter, at least 100 fibers were measured.

The chemical composition of the scaffolds was characterized using an InVia spectrometer (Renishaw, Gloucester, UK) equipped with a DM 2500 M microscope (Leica, Wetzlar, Germany) with a 50× objective. A 60 mW laser with a 532 nm wavelength and a spectral resolution of 2 cm^−1^ was used. A spectral range of 1000 to 1800 cm^−1^ was considered. The recorded spectra were deconvoluted using Gaussian line fitting in Origin 2021 software (Origin Lab, Northampton, MA, USA). The fitting parameters were used to calculate the Raman parameters, including the band position and the spectral intensity ratios (I_D_/I_G_).

X-ray photoelectron spectroscopy (XPS) was performed using a Thermo Fisher Scientific XPS NEXSA spectrometer with a monochromated Al K Alpha X-ray source working at 1486.6 eV. For the C1s and N1s high-resolution spectra, the pass energy was 50 eV, and energy the resolution was 0.1 eV. The analyzed area was 400 µm^2^. A flood gun was used for charge compensation. The samples were analyzed without precleaning.

The wettability of the fabricated scaffolds was characterized by deposition of 3 μL drops of Milli-Q water using an Easy Drop (KRÜSS, Hamburg, Germany) contact angle measurement system. Droplets were placed at different position on samples, and images were captured after 2 min disposition of each drop. Studies were carried out immediately after coating deposition and after 1, 3 and 6 months.

The cytotoxicity of the fabricated scaffolds was analyzed using a mouse embryonic fibroblast 3T3-L1 cell line. The experiment was conducted using a previously reported protocol [11].

The obtained data were statistically analyzed with Prism software (GraphPad, San Diego, CA, USA). A Kruskal-Wallis test was used for the average fiber diameter measurements and water contact angle results, whereas a Mann–Whitney test was applied to the results of cell studies. The p value was set to <0.05 for both tests.

## 3. Results and Discussion

**Figure 1:**
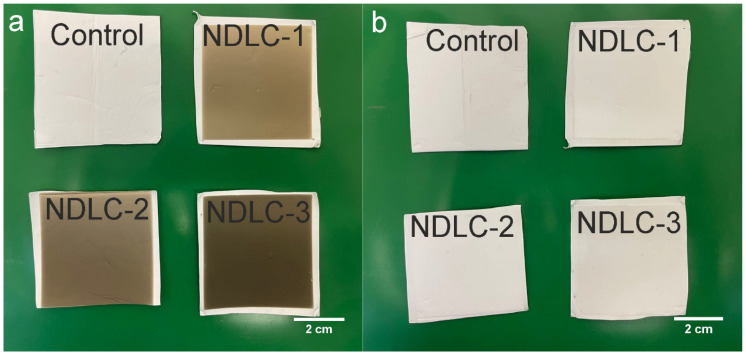
Front (**a**) and back (**b**) sides of the modified and unmodified materials.

Photographs of the front and back sides of the scaffolds after deposition of the DLC coating under various nitrogen pressure conditions are presented in Figure 1. Both sides of the control PCL scaffold were homogeneously white. Following the deposition of DLC coatings, the front side of the scaffold turned gray and became darker with increasing nitrogen pressure during the deposition process (Figure 1).

No melts or burns were observed on the front side of the scaffolds; thus, selected DLC deposition regimes preserved the scaffold macrostructure. The back side of the scaffolds remained white, demonstrating that a single-side modification of the scaffold is possible.

**Figure 2:**
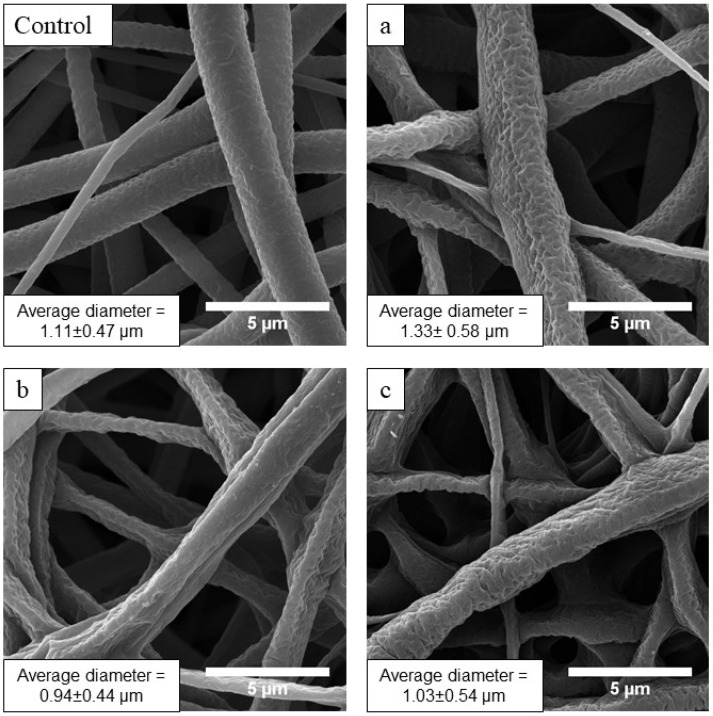
SEM images and average diameters of fabricated scaffolds fibers (×15,000 magnification)**. Control**—image of the control sample, (**a**–**c**)—images of the samples coated under 5 × 10^−3^, 5 × 10^−2^ and 5 × 10^−1^ Pa nitrogen pressure, respectively.

SEM images of the fabricated scaffolds are presented in Figure 2. The control scaffold is made up of cylindrical fibers with an average diameter of 1.00 ± 0.51 µm. Regardless of the nitrogen pressure in the chamber, no defects (fiber melting and break) or statistically significant changes in the average diameter of the fiber were observed (Figure 2). Thus, the formation of DLC coating using selected regimes preserves microstructure of the PCL electrospun scaffold.

**Figure 3:**
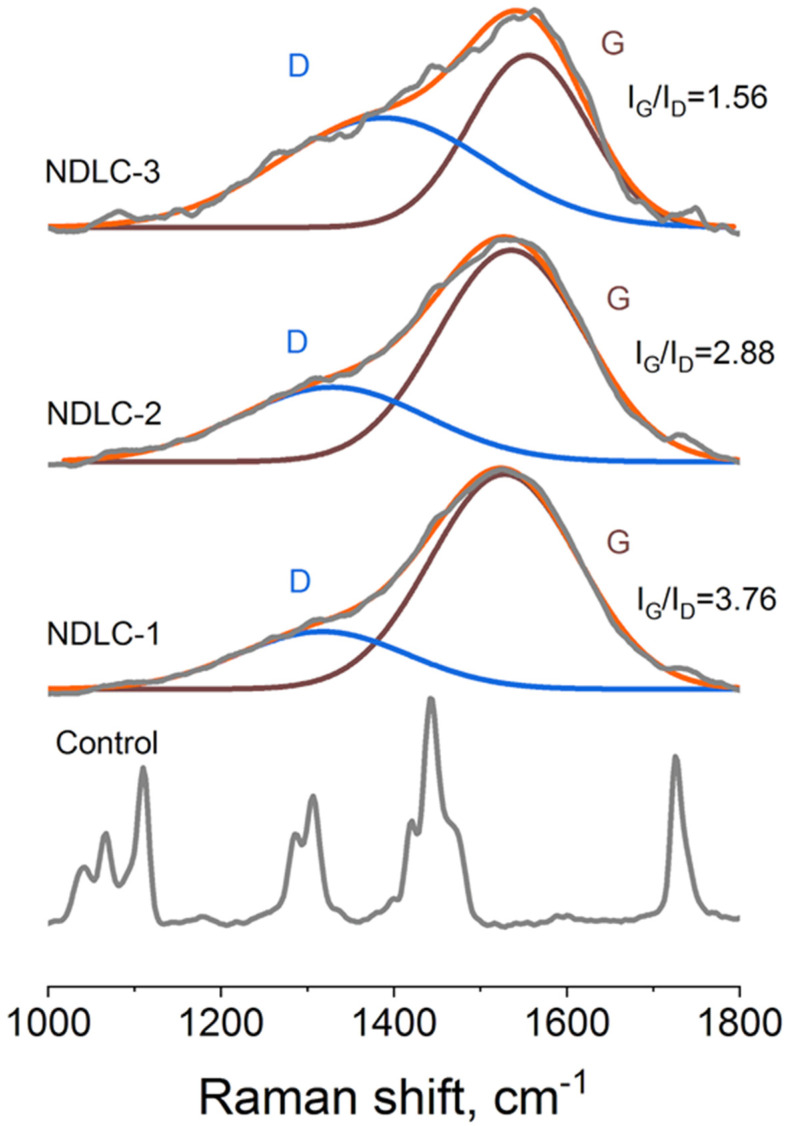
Normalized Raman spectra of the control and modified scaffolds. Blue line—peak of the disordered form of carbon (D), wine line—peak of the graphite form of carbon (G), orange line—fitted spectrum, grey line—experimental spectrum.

Normalized Raman spectra of the fabricated scaffolds are presented in Figure 3. In the 1000–1800 cm^−1^ range of the control sample spectra, specific PCL peaks were observed: 1723 cm^−1^—ν(C=O), 1441 and 1416 cm^−1^—δ(CH_2_), 1305 and 1284 cm^−1^—τ(CH_2_), 1109 and 1065 cm^−1^—ν(C–C), 913 cm^−1^—ν(C–COO) [12]. In the spectra of all DLC-coated samples, a wide peak (corresponding to disordered (D) and graphite (G) forms of carbon [13,14]) was observed (Figure 3). With increased nitrogen pressure in the chamber, a shift of the D peak from 1317 to 1387 cm^−1^ and an increase in its intensity were observed. Thus, the increase in nitrogen pressure during the deposition process resulted in a decrease in the I_G_/I_D_ ratio in the deposited coatings (Figure 3). An increase in D peak intensity following the addition of nitrogen to the working gas was observed by Menegazzo et al. during the formation of DLC coatings using the pulsed laser deposition method [15]. These observations were explained by the enlarged sp^2^ clusters. Therefore, nitrogen pressure variation is an efficient approach for the control of DLC structure during pulsed vacuum arc deposition.

X-ray photoelectron spectroscopy was used to investigate the elemental composition of the fabricated samples. The results are presented in Table 1.

The elemental composition of the pristine scaffold surface was presented by carbon (77.0 ± 2.0 at. %) and oxygen (23.0 ± 1.0 at. %), the elements present in PCL [16]. Nitrogen was not detected in the control sample. Nitrogen content in the coating formed under minimal nitrogen pressure (5 × 10^−3^ Pa) was found at 1.7 ± 0.8 at. % and increased to 16.1 ± 0.9 at. % with further increase in nitrogen pressure.

**Figure 4:**
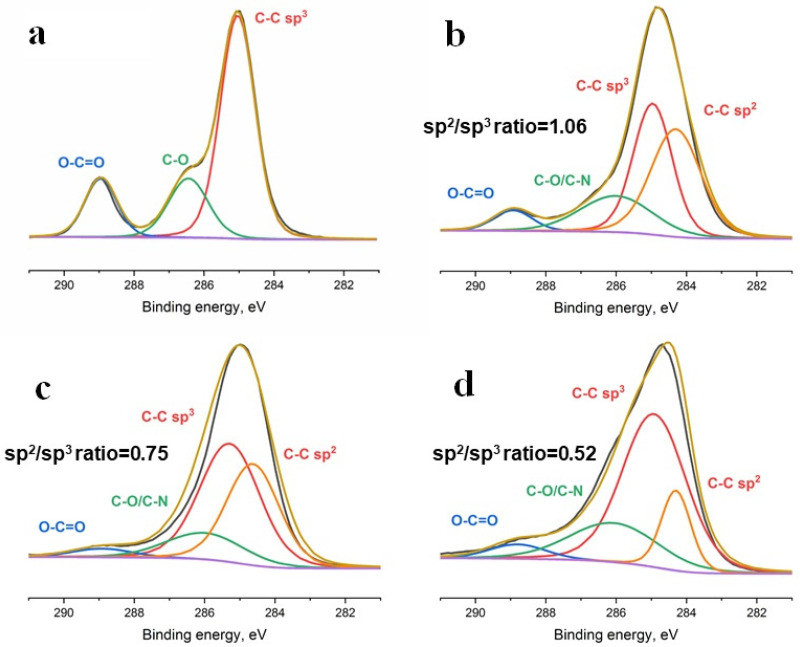
C1s spectra of the control and modified scaffolds. (**a**)—spectrum of the control sample, (**b**–**d**)—spectra of the samples coated under 5 × 10^−3^, 5 × 10^−2^ and 5 × 10^−1^ Pa nitrogen pressure, respectively.

Deconvoluted C1s spectra of the fabricated samples are shown in Figure 4. The control sample spectrum presented with peaks specific to PCL: O–C=O (289.0 eV), C–O (286.4 eV) and C–C sp^3^ (285.0 eV) [16]. On the spectra of the coated samples, the peaks corresponding to N-doped DLC coatings (O–C=O (289.0 eV), C–O/C–N (286.0 eV), C–C sp^3^ (285.0 eV) and C–C sp^2^ (284.3 eV)) were observed [13]. Deconvolution of C1s spectra revealed a decrease in the sp^2^/sp^3^ ratio from 1.06 to 0.52 with an increase in nitrogen pressure (Figure 4). Thus, XPS results confirm the tendency demonstrated by Raman spectroscopy.

**Figure 5:**
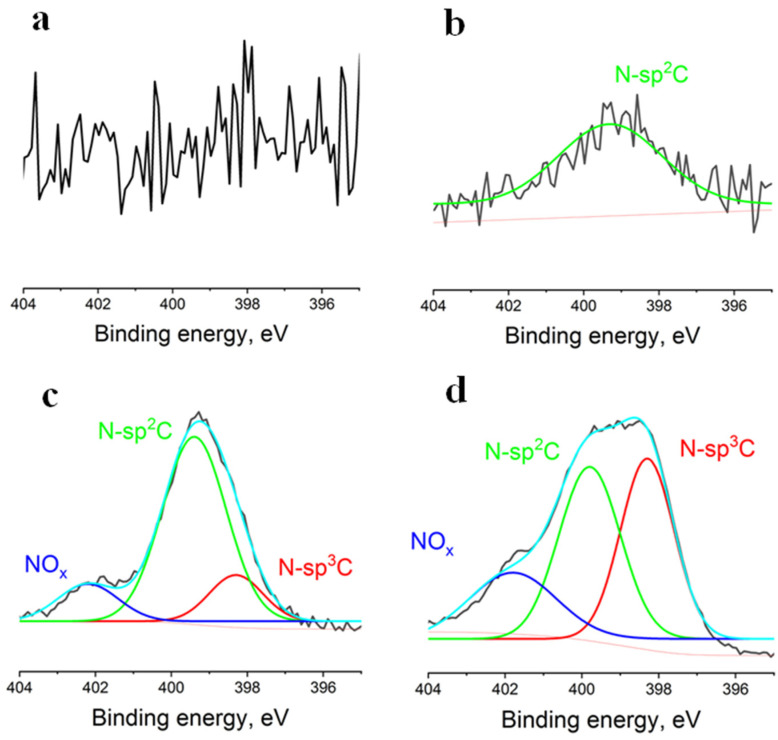
N1s spectra of the control and modified scaffolds. (**a**)—spectrum of the control sample, (**b**–**d**)—spectra of the samples coated under 5 × 10^−3^, 5 × 10^−2^ and 5 × 10^−1^ Pa nitrogen pressure, respectively.

N1s spectra of the fabricated samples are presented in Figure 5. No peaks were detected on the spectrum of the pristine PCL scaffold. However, on the spectrum of the sample coated under minimum nitrogen pressure, a peak corresponding to nitrogen bonded to sp^2^-hybridized carbon (399.3 eV) was observed. With further increase in nitrogen pressure, a peak corresponding to nitrogen bonded to sp^3^-hybridized carbon (398.3 eV) and a high-energy peak apparently corresponding to nitrogen oxides (401.8 eV) [17] appeared. Water and air adsorbed on the surface of the pristine scaffolds before coating deposition may serve as oxygen source in that case.

The results of the study of long-term hydrophilicity of the scaffolds are presented in Table 2. The surface of the control scaffold was hydrophobic (water contact angle of 126 ± 2°), which is typical of PCL scaffolds [18]. The deposition of the DLC coating under a nitrogen pressure of 5 × 10^−3^ Pa did not have a statistically significant effect on the surface wetting. A further increase in nitrogen pressure in the chamber resulted in a decrease in water contact angle, with a nitrogen pressure of 5 × 10^−1^ Pa providing a sixfold decrease compared to the control. The observed effect may be caused by numerous factors. Ostrovskaya et al. reported a decrease in water contact angle of various carbon surfaces with increased sp^3^-hybridized carbon content [19]. Subramanian et al. demonstrated uneven effect of various dopants (fluorine, silicon and nitrogen) on the wetting of DLC coatings [20]. Kashyap et al. showed an increase in wettability with increased roughness of DLC coatings [21]. Thus, it is complicated to reveal the key factor contributing to an enhanced hydrophilicity of the fabricated materials. Importantly, the high hydrophilicity of the coating deposited under a nitrogen pressure of 5 × 10^−1^ Pa did not vary significantly during the 6 months of the experiment (Table 2).

Images of the cells cultured with the extracts of the obtained membranes collected on day 5 with DLC coatings deposited under various conditions are presented in Supplementary Figure S1. The results of cell viability studies in the sample extracts are presented in Table 3. The viability of the cells cultured in control DMEM was taken as 100%. Cells demonstrated high viability and formed a confluent layer after 72 h of cultivation. The PCL scaffold extract did not contain any toxic compounds, as cell viability in that group did not differ from that of the control medium.

The viability of cells cultured in DLC-coated sample extracts for 24 h did not differ from that of the control group and was around 100 %, regardless of the deposition regime. Moreover, as for the cells cultured in control medium, a confluent layer was formed after 72 h of cultivation. No statistically significant differences were observed between the groups during the experiment. Thus, DLC coatings formed on the surface of PCL electrospun scaffolds by means of pulsed vacuum arc deposition in a nitrogen atmosphere contain no toxic compounds, indicating their prospective for biomedical applications.

## 4. Conclusions

Diamond-like coatings were deposited on the surface of electrospun poly(ε-caprolactone) scaffolds using pulsed vacuum arc deposition with sputtering of graphite target in a nitrogen atmosphere. Single-side hydrophilization (water contact angle around 22°) of the materials was achieved for up to 6 months. By means of Raman and X-ray photoelectron spectroscopy, it was established that variation of nitrogen pressure is an efficient tool to adjust the sp^2^/sp^3^ carbon ratio in the coating without disrupting or altering scaffold morphology. Investigation of chemical structure of the deposited coatings showed that an increase in nitrogen pressure in the chamber leads to the formation of “carbon–nitrogen” and “nitrogen–oxygen” bonds. In vitro experiments did not reveal any cytotoxic activity of the fabricated material extracts against fibroblasts. The reported studies demonstrate the prospects of the pulsed vacuum arc deposition technique for long-term hydrophilization of electrospun poly(ε-caprolactone) tissue engineering scaffolds.

## Figures and Tables

**Table 1:** Elemental composition of the control and modified scaffolds.

Sample	Carbon, at. %	Oxygen, at. %	Nitrogen, at. %
Control	77.0 ± 2.0	23.0 ± 1.0	-
NDLC-1	85.0 ± 1.0	14.0 ± 1.0	1.7 ± 0.8
NDLC-2	86.3 ± 1.3	8.8 ± 1.0	4.9 ± 0.8
NDLC-3	76.2 ± 0.9	9.9 ± 1.3	16.1 ± 0.9

**Table 2:** Water contact angles of the fabricated samples.

Sample	Water Contact Angle, Deg			
	Immediately after Deposition (n = 5)	1 Month after Deposition (n = 5)	3 Months after Deposition (n = 5)	6 Months after Deposition (n = 5)
Control	126 ± 2	126 ± 3	130 ± 3	133 ± 2
NDLC-1	107 ± 6	110 ± 3	115 ± 2	121 ± 6
NDLC-2	89 ± 2 *	91 ± 5 *	95 ± 5 *	107 ± 7 *
NDLC-3	22 ± 3 *	23 ± 4 *	21 ± 3 *	21 ± 3 *

* *p* < 0.05 compared to the control (Kruskal–Wallis test).

**Table 3:** Viability of fibroblasts during cultivation in extracts from fabricated samples.

Sample	Cultivation Time					
	24 h		72 h		120 h	
	Cell Viability, %	Cell Density, cells/mm^2^	Cell Viability, %	Cell Density, cells/mm^2^	Cell viability, %	Cell Density, cells/mm^2^
Control culture medium	100 ± 3	210 ± 11	100 ± 3	1148 ± 88	100 ± 7	1450 ± 75
Control	99 ± 6	215 ± 14	100 ± 6	1130 ± 81	97 ± 4	1419 ± 57
NDLC-1	96 ± 5	215 ± 15	98 ± 8	1128 ± 58	100 ± 7	1431 ± 105
NDLC-2	102 ± 4	207 ± 12	101 ± 4	1110 ± 90	100 ± 6	1458 ± 79
NDLC-3	101 ± 4	223 ± 36	99 ± 2	1132 ± 118	96 ± 7	1425 ± 64

## Data Availability

Not applicable.

## Supplementary Materials

The following supporting information can be downloaded at: https://www.mdpi.com/article/10.3390/membranes12111080/s1, Figure S1. Images of the fluorescently labeled fibroblasts after 24, 72 and 120 h of cultivation with sample extracts.
