# Supplementary material for: Pulsed Vacuum Arc Deposition of Nitrogen-Doped Diamond-like Coatings for Long-Term Hydrophilicity of Electrospun Poly(ε-caprolactone) Scaffolds
Source: Membranes (Basel). 2022 Oct 31;12(11):1080. doi: 10.3390/membranes12111080 (PMC9695898; doi:10.3390/membranes12111080)
Supplement: Supplementary file 1 [file membranes-12-01080-s001.zip › membranes-1965422-supplementary.pdf]

Supplementary materials

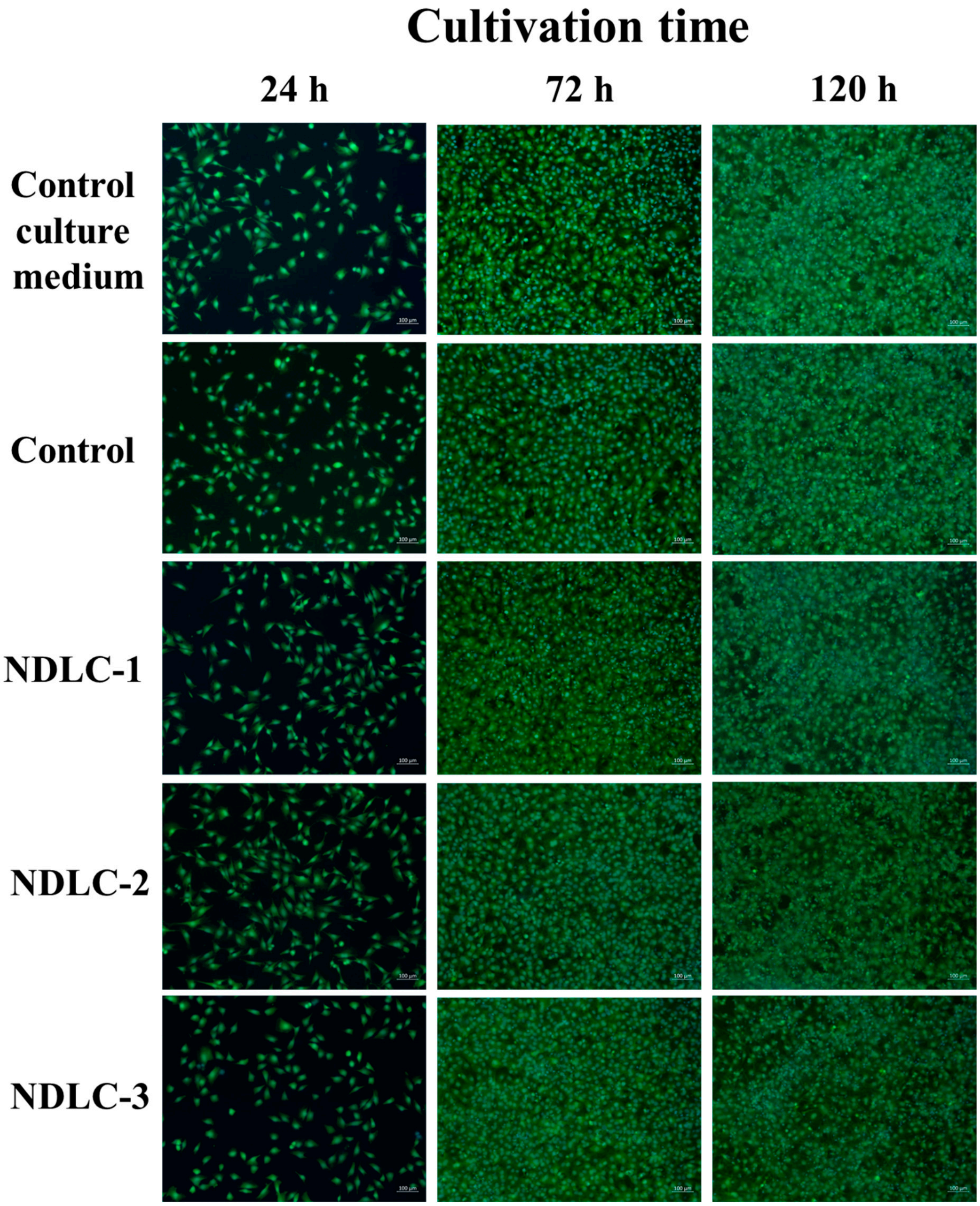

Figure S1. Images of the fluorescently labeled fibroblasts after 24, 72 and 120 h of cultivation with sample extracts.
